# Development of an *in vitro* drug sensitivity assay for *Trichuris muris* first-stage larvae

**DOI:** 10.1186/1756-3305-6-42

**Published:** 2013-02-22

**Authors:** David Wimmersberger, Lucienne Tritten, Jennifer Keiser

**Affiliations:** 1 Department of Medical Parasitology and Infection Biology, Swiss Tropical and Public Health Institute, P.O. Box, Basel, CH–4002, Switzerland; 2 University of Basel, Basel, CH–4003, Switzerland

**Keywords:** *Trichuris muris*, Nitazoxanide, Levamisole, Oxantel pamoate, *In vitro* assay, Resazurin, Fluorescein-albumin, Calcein AM, Ethidium homodimer-1

## Abstract

**Background:**

Trichuriasis represents a major public health problem in the developing world and is regarded as a neglected disease. Albendazole and mebendazole, the two drugs of choice against trichuriasis display only moderate cure rates, hence alternative drugs are needed. To identify candidate compounds, *in vitro* drug sensitivity testing currently relies on the adult *Trichuris muris* motility assay. The objective of the present study was to develop a simple and cost-effective drug sensitivity assay using *Trichuris muris* first-stage larvae (L1).

**Methods:**

Several potential triggers that induce hatching of *T. muris* were studied, including gastrointestinal enzymes, acidic environment and intestinal microflora. Next, optimal culture conditions for *T. muris* L1 were determined assessing a wide range of culture media. *T. muris* L1 were incubated in the presence of mebendazole, ivermectin, nitazoxanide, levamisole or oxantel pamoate at 37°C. The viability of the parasites was evaluated microscopically after 24 hours. The usefulness of fluorescent markers (resazurin, calcein AM, ethidium homodimer-1 or fluorescein-conjugated albumin) in drug sensitivity testing was also assessed.

**Results:**

The established L1 motility assay provided accurate and reproducible drug effect data *in vitro.* IC_50_ values for oxantel pamoate, levamisole and nitazoxanide were 0.05, 1.75 and 4.43 μg/mL, respectively. Mebendazole and ivermectin failed to show any trichuricidal effect on L1. No correlation was found between data from the four fluorescent markers and the comparative motility assay.

**Conclusions:**

The motility assay based on L1 was found suitable for drug sensitivity screening. It is rather simple, cost-effective, time-saving and sustains medium-throughput testing. Furthermore, it greatly reduces the need for the animal host and is therefore more ethical. None of the viability markers assessed in this study were found to be satisfactory.

## Background

Human trichuriasis, caused by the gut-dwelling soil-transmitted helminth species *Trichuris trichiura*, represents a major public health problem in the developing world since an estimated 604–795 million people are infected worldwide [1]. *T. trichiura* infections are responsible for the loss of 349,000–1,061,000 disability-adjusted life years (DALYs) per year [2]. Particularly affected, school- and preschool-aged children are confronted with the largest morbidity burden [3]. Mild infections are often asymptomatic, however, in severe and chronic infections patients can suffer from dysentery, anemia and impairment of physical and mental development [3]. The drugs recommended by the World Health Organization [4], albendazole and mebendazole, have been in use for decades and display only moderate cure rates when administered as single doses [5-7]. Although this has not arisen as a problem yet, potential emergence of drug resistance is of concern [5,6]. Thus, there is a need to find novel anthelmintic drugs for the treatment of this disease.

For drug testing purposes, a user-friendly and inexpensive *in vitro* assay is desirable to accurately determine the drug sensitivity of the worms as a first screen filter. Using the mouse whipworm *T. muris*[8,9], the current assay of choice is the adult motility assay [10-12]. It is well established and reliable but suffers from significant drawbacks: it is expensive, time-consuming and necessitates the sacrifice of the murine host and therefore, is not suitable for medium- to high-throughput screening in its actual form.

The aim of the present study was to develop a simple, cost-effective and time-saving alternative to the drug sensitivity assay currently in use. After excluding potential hatching triggers such as intestinal enzymes and gastric pH, we exploited the ability of first-stage larvae (L1) to hatch following exposure of *T. muris* eggs to intestinal bacteria, such as *Escherichia coli*[13]. Subsequently, optimal culture conditions for L1 were determined and the currently used adult motility assay was adapted to L1. The trichuricidal effects of three standard anthelmintics (mebendazole, ivermectin and levamisole) were studied and compared to two potential alternative drugs (nitazoxanide and oxantel pamoate).

In addition, we assessed four potential viability markers, resazurin (Alamar Blue^®^), fluorescein-albumin isothiocyanate conjugate (feeding inhibition assay), calcein acetoxymethyl ester (AM) and ethidium homodimer-1 (both from LIVE/DEAD^®^ cell viability kit).

Resazurin is a colorimetric and fluorescent dye that has been successfully used in *in vitro* drug sensitivity assays for several parasitic species such as African trypanosomes and more recently for adult *T. muris*[10,14]. The underlying principle of this dye is the enzymatic reduction of the non-fluorescent molecule resazurin to the fluorescent resorufin by viable cells. The feeding inhibition assay has been used to analyze the feeding behavior of *Ancylostoma* spp. larvae [15-17]. Viable parasites might ingest medium containing the fluorescent molecule (bovine albumin conjugated to fluorescein), which allows the discrimination from dead parasites by microscopical observation of the stained intestinal tract of the worms [12,17]. Finally, we assessed the two-compound LIVE/DEAD^®^ cell viability assay which is designed to dye viable as well as dead mammalian cells and has been used in *Leishmania* research [18,19]. The kit consists of two different dyes: the green-fluorescent calcein AM indicates intracellular esterase activity and thus stains live cells and the red-fluorescent ethidium homodimer-1 (EthD-1) binds to DNA of dead cells presenting a disrupted plasma membrane.

## Methods

### Drugs, chemicals and media

Ivermectin and mebendazole were purchased from Sigma-Aldrich (Buchs, Switzerland). Levamisole and oxantel pamoate were purchased from Fluka (Buchs, Switzerland). Nitazoxanide was obtained from Laboratoria Wolfs (Zwijndrecht, Belgium). Drug stocks were made in 100% DMSO (dimethyl sulfoxide, Fluka, Buchs, Switzerland) at a concentration of 10 mg/mL and stored at 4°C pending use.

Resazurin (resazurin sodium salt, 125 mg/L), amphotericin B (250 μg/mL), fluorescein-albumin isothiocyanate conjugate lipase (from porcine pancreas), Luria Broth (25 g/L), penicillin-streptomycin (10,000 units penicillin + 10 mg/mL streptomycin) and glucose (anhydrous, 96%) were purchased from Sigma-Aldrich (Buchs, Switzerland). Bile salts, ciprofloxacin and pepsin (from porcine gastric mucosa) were purchased from Fluka (Buchs, Switzerland). Dulbecco's modified eagle medium (DMEM), Hanks’ balanced salt solution (HBSS), Medium 199, Minimum essential medium (MEM) and RPMI 1640 were purchased from Gibco (Basel, Switzerland). Fetal calf serum (FCS) was obtained from Connectorate (Dietikon, Switzerland). LIVE/DEAD^®^ cell viability kit (L-3224) was purchased from Invitrogen (Carlsbad, CA, USA). RPMI medium was prepared using 10.44 g RPMI 1640, 5 g albumax H (Gibco), 5.94 g HEPES (Sigma-Aldrich) and 2.1 g sodium bicarbonate (Sigma-Aldrich) in 1 L deionized water.

### Parasites and bacteria

The life cycle of *T. muris* is maintained at the Swiss TPH since 2010 as described elsewhere [10]. Briefly, unembryonated eggs released in the feces of infected mice were isolated and purified through flotation with saturated NaCl (359 g/l in deionized water) and cultured in tap water at room temperature for at least eight weeks. Embryonation of eggs was monitored microscopically.

*Escherichia coli* stem BL-21 was ordered from New England Biolabs (Ipswich, MA, USA). The bacteria were stored in Luria Broth (LB) medium and 30% glycerin at −80°C. To provide bacteria for the hatching process, a pipette tip of the frozen *E. coli* suspension was dipped in LB medium (25 g/L) and incubated for 12 to 24 hours at 37°C.

### *In vitro* studies

#### Hatching

Several potential triggers of the hatching process of *T. muris* larvae mimicking the intestinal environment were studied. In all experiments eggs were evaluated microscopically (magnification: 40x) after incubation in the presence of the trigger candidate.

##### Enzymes

*T. muris* egg suspensions were incubated together with the gastric enzyme pepsin (3.2 g/L; pH: 2) or the intestinal enzyme lipase (20 g/L; pH: 7.94; coenzyme: bile salts) at 37°C for up to 24 hours. The combination of the two enzymes was studied, too, incubating eggs first in the presence of lipase (20 g/mL) for 4 hours at 37°C and subsequently adding pepsin (1.6 g/mL) for another 24 hours of incubation at 37°C. Each of these experiments was performed twice independently.

##### Acidic conditions

*T. muris* eggs were exposed to 0.9% NaCl solution with acidic pH values of 1, 1.5, 2, 2.5 and 3 at 37°C mimicking the mouse stomach conditions [20]. The experiment was performed twice independently.

##### Exposure to intestinal bacteria

*T. muris* eggs (1200/mL) were incubated in RPMI medium (containing 12.5 μg/mL amphotericin B, 500 units/mL penicillin, 500 μg/mL streptomycin) in the presence of viable *E. coli* (10^7^-10^8^ cells/mL) at 37°C for 4 hours. Bacterial concentration was determined by optical density measurement at 600 nm using the formula: OD x 5 x 10^8^ = bacterial cells/mL. The hatching rate, or proportion of hatched larvae in each well, was determined microscopically after 4 hours (magnification: 40x).

#### Optimal culture conditions

The viability of *T. muris* L1 was tested in 5 different liquid culture media (RPMI, MEM, DMEM, Medium 199 and HBSS). All media contained 12.5 μg/mL amphotericin B, 500 units/mL penicillin and 500 μg/mL streptomycin. Survival rates of the larvae were evaluated microscopically after 24, 48 and 72 hours of incubation at 37°C following stimulation of the larvae with hot tap water (≈ 70°C). Motile L1 were considered as viable and immotile L1 as dead. The addition of a supplement to the basic RPMI medium namely 20 g/L glucose or 5% FCS was also assessed. This experiment was performed twice in triplicate for each medium with an average of 30 L1 per well.

Finally, incubation of L1 at 37°C was compared to room temperature (approximately 24°C). The survival rates of the newly hatched larvae in RPMI medium were examined microscopically after 24, 48, 72, 96 and 120 hours. This experiment was conducted twice in triplicate using approximately 40 worms per well.

#### Motility assay

The assay methodology was adapted from the assays described for hookworm and *Strongyloides* spp. [21] and for adult *T. muris*[10-12]. Mebendazole, levamisole, ivermectin, nitazoxanide and oxantel pamoate were tested at concentrations of 1, 10 and 50 μg/mL. Oxantel pamoate was additionally tested at concentrations of 0.1 and 0.01 μg/mL. Wells containing parasites exposed to 0.5% DMSO served as controls. The assay was performed in 96-well plates containing approximately 25 worms per well in a total volume of 100 μL (50 μL containing L1 + 50 μL drug solution). The well plates were incubated for 24 hours at 37°C. The motility was then analyzed by stimulating the parasites with hot tap water (≈ 70°C), using a light microscope (magnification: 40x). A binary scale was used to qualify viability, discriminating live from dead larvae: “0” = no sign of motion = dead and “1” = motion observed = alive. The percentage of dead larvae was established for each well. For each drug and concentration, the assay was conducted in 5 wells, three times independently.

#### Viability marker assays

##### Resazurin assay

Twenty μL of resazurin were added to wells containing 100 μL of *T. muris* L1 (approximately 50 per well) and/or *E. coli* suspensions (medium: RPMI 1640 containing 12.5 μg/mL amphotericin B, 500 units/mL penicillin and 500 μg/mL streptomycin). The trichuricidal effect of nitazoxanide was assessed and compared to live controls (viable parasites exposed to 0.5% DMSO) and larvae killed with 70% ethanol. Fluorescent emission was measured at 560 nm (excitation) and 590 nm (emission) after 12, 24, 36 and 48 hours using a spectrofluorometer (SpectraMax, Gemini XS, Molecular Devices, UK). The antibiotic ciprofloxacin (500 μg/mL) was used to eliminate bacterial contamination of the medium after completion of hatching. The experiment was repeated three times.

##### Feeding inhibition assay

One hundred μL of a 1 mg/mL fluorescein-albumin isothiocyanate conjugate solution (solubilized in RPMI 1640) were added to wells containing 100 μL of *T. muris* L1 (approximately 50 larvae per well) in RPMI 1640 (containing 12.5 μg/mL amphotericin B, 500 units/mL penicillin, 500 μg/mL streptomycin and 500 μg/mL ciprofloxacin). The well plate was incubated at 37°C and after 3 hours the larvae were centrifuged (14,000 rpm) and washed three times with phosphate buffered saline (PBS). The larvae were analyzed after 3 and 24 hours using an inverted fluorescence microscope (Carl Zeiss, Germany, magnification: 100–200x, excitation: 450–490 nm, emission: 520 nm) [12]. Emissions from motile larvae were compared to those from immotile larvae. The experiment was conducted twice independently.

##### LIVE/DEAD^®^ cell viability assay

Newly hatched *T. muris* L1 were incubated at different concentrations (from 25 to 500 parasites per well) at 37°C in 100 μL RPMI 1640 (containing 12.5 μg/mL amphotericin B, 500 units/mL penicillin, 500 μg/mL streptomycin and 500 μg/mL ciprofloxacin). After 16 hours, 100 μL of a calcein AM/ethidium homodimer-1 solution (8 μM EthD-1 and 4 μM calcein AM solubilized in RPMI 1640) were added to each well. The plate was analyzed after 30 minutes, 1, 2, 3, 4 and 24 hours using a spectrofluorometer (SpectraMax, Gemini XS, Molecular Devices, UK; calcein AM: excitation: 485 nm, emission: 525 nm; EthD-1: excitation: 525 nm, emission: 645 nm). The emission values were adjusted to the background noise defined as the average signal produced by wells containing *E. coli* only. Emissions from motile larvae were compared to those from immotile larvae. Using 100 L1 per well, an assay was performed with nitazoxanide and levamisole at concentrations of 1, 10 and 50 μg/mL (0.5% DMSO served as a control sample). Samples were analyzed using a fluorescence microscope using texas red- (excitation: 577 nm; emission: 620 nm) and L5-filters (excitation: 480 nm; emission: 527 nm). The assay was performed three times independently.

### Statistical analysis

All data sets were analyzed by OpenOffice (Apache Software Foundation, 2010). Means (± standard deviations) of motilities and emissions were calculated from repeated tests. Motility differences between drug treatments and controls were tested for significance using the Fisher’s exact test in R (R 2.12.0, R Development Core Team, 2012). IC_50_ values of the motility assay were calculated based on median effect principle using the CompuSyn software (Ting-Chao Chou and Nick Martin; ComboSyn, Inc., 2007) [22]. These were defined as the concentration of a drug required to decrease the mean worm motility to 50%. The r value is the linear correlation coefficient of the median-effect plot. It illustrates the goodness of fit, and thus the accuracy of the IC_50_ value.

## Results

### Hatching

#### Enzymes

After 24 hours in a pepsin solution (3.2 g/L; pH: 2), embryonated *T. muris* eggs were phenotypically still intact. No digestive degradation of the eggshell could be observed, nor had hatching been induced. Embryonated *T. muris* eggs exposed to lipase (20 g/L; pH: 7.94), were found intact after 24 hours and no hatching had taken place. Similarly, the addition of pepsin (1.6 g/L) after 4 hours to the lipase solution did not show any effect after 24 hours of incubation at 37°C.

#### Acidic conditions

No alteration of the eggshell could be observed microscopically after 2 hours of incubation at any pH value studied at 37°C and no larva was observed to hatch.

#### Intestinal microflora

**Figure 1:**
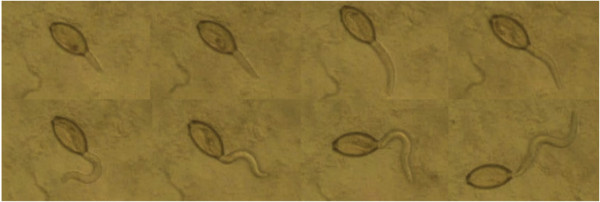
**Hatching process of *T. muris *after exposure to *E. coli *(duration: approximately 2 minutes; magnification: 40x).**

Exposing *T. muris* eggs to an *E. coli* suspension induced hatching of approximately 70% of the eggs after 2–4 hours of incubation at 37°C. Figure 1 shows the hatching process of a larva. The larvae moved spontaneously in a sinusoidal motion. Bacterial concentrations of 5x10^7^/mL and beyond successfully induced hatching in 80% of the tests, at a rate ranging between 60 and 80%. The hatching rate did not increase with *E. coli* concentrations over 5x10^7^/mL.

### Optimal culture conditions

#### Media

In RPMI medium, 92.1% (± 1.2%) of the larvae survived over 72 hours. The presence of 20 g/L glucose or 5% FCS did not improve the survival rates compared to RPMI medium alone (79.5% ± 11.3% and 81.5% ± 3.0%, respectively after 72 hours). In MEM and DMEM larvae showed maximal survival rates of 23.8% and 6.5%, respectively, after 24 hours. Similarly, larvae incubated in HBSS and in Medium 199 displayed poor survival rates after 72 hours (24.8% and 15.1%, respectively). The basic RPMI medium was used in all subsequent hatching and drug sensitivity tests.

#### Temperature

L1 survived for at least 72 hours at 37°C in RPMI medium. After 24 hours at room temperature all larvae were dead.

### Survival rates in RPMI medium

**Figure 2:**
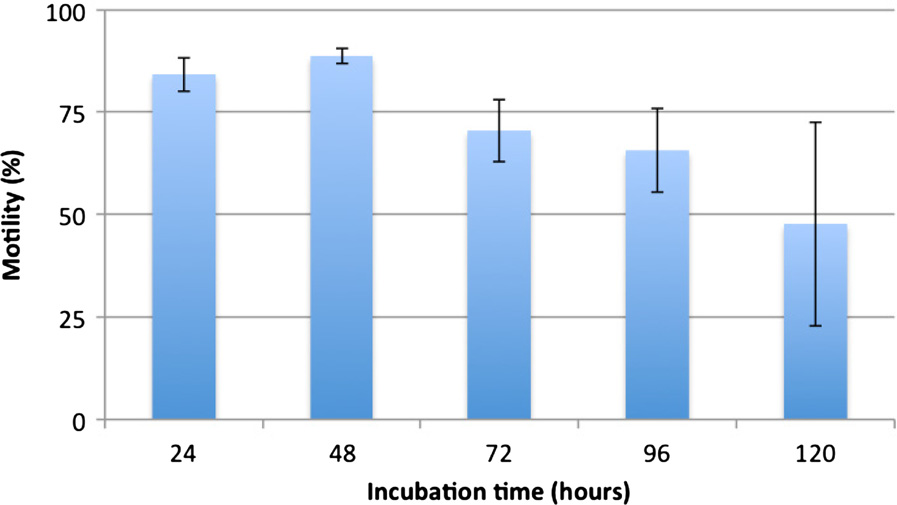
**Survival rates (motility in percent) of *T. muris *L1 after 24, 48, 72, 96 and 120 hours.** Data are based on two individual experiments, each performed in triplicate.

Since RPMI medium was found to be the ideal medium for maintenance of *T. muris* L1 *in vitro*, behavior of L1 in this medium was studied in greater detail. Figure 2 shows the survival rates of L1 after 24, 48, 72, 96 and 120 hours in RPMI medium at 37°C. Until 48 hours after hatching, the larvae showed survival rates over 80% (84.3% ± 4.0% after 24 hours and 88.8% ± 1.8% after 48 hours). After 72 and 96 hours, survival rates decreased constantly until eventually falling below 50% after 120 hours (70.4% ± 7.6%, 65.6% ±10.2% and 47.6% ± 24.8%, respectively).

### Motility assay

**Figure 3:**
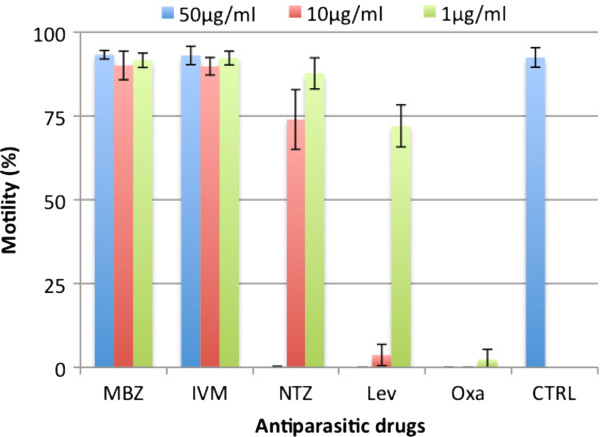
**Survival rates (motility in percent) of *T. muris *L1 exposed to different drugs at concentrations of 1, 10 and 50 μg/mL for 24 hours.** MBZ: mebendazole; IVM: ivermectin; NTZ: nitazoxanide; LEV: levamisole; OXA: oxantel pamoate; CTRL: control; 0.5% DMSO. Data are based on three individual assays, each performed in quintuplicate.

As shown in Figure 3, the antiparasitic drug nitazoxanide as well as the anthelmintics oxantel pamoate and levamisole showed a much greater effect *in vitro* than the two standard drugs mebendazole and ivermectin. Drug concentrations of 50 μg/mL of mebendazole or ivermectin had no trichuricidal effect, as illustrated by the average survival rates of 93.3% (± 2.9%) and 93.1% (± 1.3%), respectively, and did not differ significantly from the controls (92.5%; ± 2.9%; all p > 0.1). The same concentration of nitazoxanide, levamisole or oxantel pamoate was lethal in all three cases (0% survival; all p < 0.005). Nitazoxanide displayed a lower effect at concentrations of 10 μg/mL and below: 74.0% survival at 10 μg/mL (± 8.9%; p < 0.005) and 87.7% at 1 μg/mL (± 4.6%; p = 0.046). Fifty and 10 μg/mL levamisole decreased survival by >90% while 72.0% (± 6.3%) of the larvae survived after exposure to 1 μg/mL (p < 0.005). A concentration of 1 μg/mL oxantel pamoate killed almost all of the larvae (survival rate: 2.5%; ± 2.9%). Survival rates of 51.9% (± 7.8%) and 78.6% (± 11.7%) were determined for 0.1 and 0.01 μg/mL oxantel pamoate, respectively (not shown; all p < 0.005). Nitazoxanide, levamisole and oxantel pamoate displayed IC_50_ values of 4.4 (r: 0.86), 1.8 (r: 0.98) and 0.05 μg/mL (r: 0.97), respectively.

### Viability marker assays

#### Resazurin assay

**Figure 4:**
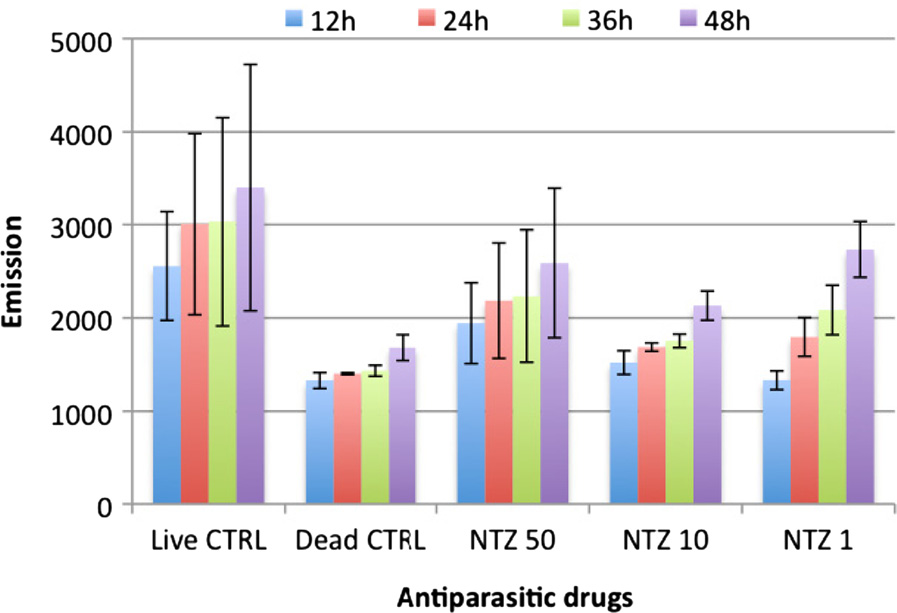
**Drug assay testing nitazoxanide using the viability marker resazurin.** Live CTRL: No drug added; Dead CTRL: Larvae killed with 70% ethanol; NTZ: nitazoxanide at concentrations of 50, 10 and 1 μg/mL. This experiment was performed three times independently in triplicate, an example is shown.

After 12 hours of incubation, wells containing viable larvae displayed higher fluorescent emissions (2557 ± 583.4) than wells containing larvae killed with 70% ethanol (1328 ± 82.6) (Figure 4). However, using nitazoxanide as test compound, a lack of correlation between the drug concentration and the signal measured was observed. For instance, wells containing parasites exposed to 1, 10 and 50 μg/mL had the following emission values: 1331 (± 98.2), 1519 (± 127.6) and 1944 (± 435.4), respectively. These findings do not correlate with the efficacy of this drug observed in the motility assay (see motility assay section). In addition, wells containing higher drug concentrations also displayed higher fluorescent emissions than wells with lower drug concentrations and the strength of the signal varied between experiments.

#### Feeding inhibition assay

Evaluation with a fluorescence microscope demonstrated that *T. muris* L1 fail to ingest significant amounts of fluorescein-containing medium since no staining was observed in their gastrointestinal tract.

#### LIVE/DEAD^®^ cell viability assay

Ethidium homodimer-1 (staining dead cells) did not show any significant fluorescent emission. The emission from the positive control wells containing motile L1 was higher than the emission of larvae exposed to oxantel pamoate. The microscopic analysis (using the texas red filter) revealed indistinguishable fluorescent emissions between viable and dead larvae.

**Figure 5:**
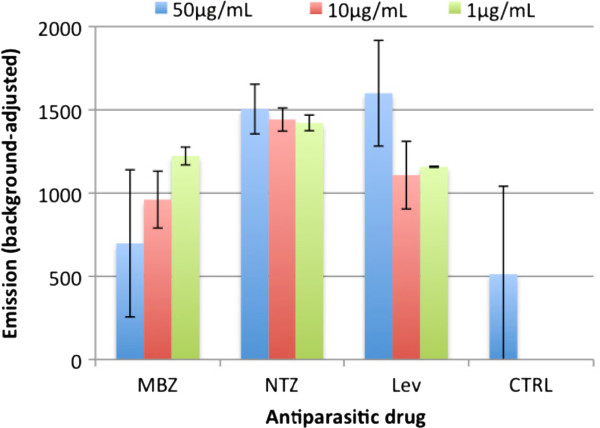
**Viability assay of live *T. muris *L1 at different concentrations (25, 50, 75, 100 and 150 parasites per well) compared to dead L1.** Y-axis shows background-adjusted fluorescent emission of calcein AM. Experiment performed twice independently in triplicate, an example is shown.

In contrast to EthD-1, worms incubated with calcein AM showed a significant signal after 1 hour already. Figure 5 shows the background-adjusted emissions of viable *T. muris* L1 compared to dead controls after 4 hours of incubation with calcein AM. The measured signals failed to correlate with the number of parasites in each well. For instance, wells containing 25 viable L1 had a similar emission as wells containing 150 larvae (1260 ± 285.8 compared to 1187 ± 577.9). Emission values of wells containing dead parasites ranged from 29 (± 75.4) for 50 worms to 807 (± 197.6) for 150 worms. Furthermore, microscopic observation of the L1 staining was not found to be sufficient to discriminate live from dead larvae. In addition, drug-treated worms revealed higher signals than control worms. For instance, wells containing worms exposed to lethal concentrations of 50 μg/mL nitazoxanide and levamisole displayed fluorescent emissions of 1505 (± 149.2) and 1600 (± 317.5), respectively, whereas only 513 (± 528.0) was measured for control worms. The signals emitted did not correlate with the motile activity of the larvae observed in the motility assay, where nitazoxanide and levamisole (50 μg/mL) reduced the viability of L1 by 100%.

## Discussion

Trichuriasis affects up to one billion people in the poorest regions of the world [1,23]. The drugs currently used against this parasite have a limited efficacy and have been used for decades [5,6]. Therefore, alternative treatments must be found. The current approach to identify potential new drug candidates relies essentially on the *T. muris* murine model. The *in vitro* assay of choice, the adult motility assay is expensive, labor-intensive, time-consuming and raises ethical questions. In this context, the objective of the present study was to develop a cost-effective and time-saving alternative assay. For this purpose, we developed a simple and reliable method to induce hatching of *T. muris* embryonated eggs exploiting findings from basic research [13]. The intestinal bacterium *Escherichia coli* was identified as optimal hatching stimulus while a series of potential triggers such as acid and intestinal enzymes were excluded.

The results obtained with the motility assay based on L1 correspond to findings observed with adult *T. muris*[10,12,24]. However, generally, L1 seem to be more sensitive to the drugs studied than older *T. muris* stages. For instance, Tritten *et al.*[12] determined the IC_50_ of levamisole to be 33.1 μg/mL for L3 and 16.5 μg/mL for adult worms whereas we calculated a value of 1.75 μg/mL for L1. An IC_50_ of 2.35 μg/mL was calculated for oxantel pamoate against L4 [24], whereas only 0.05 μg/mL was found for L1 in the present study. Nonetheless, the new assay might serve as a pre-screening tool and therefore complement assays using older larval stages and adult worms that necessitate sacrifice of the animal host. Thus, the number of necessary animal hosts can be reduced and the number of compounds to be tested can be increased.

Some characteristics of this new method are listed in Table 1 and compared to the assay for adult worms. The motility assay designed for adult *T. muris* was run over 72 hours and drug concentrations ranging from 50 to 200 μg/mL were used [10-12]. Since L1 were found to be more sensitive to the drugs tested, we performed the drug assay with lower drug concentrations (0.01 to 50 μg/mL) and microscopic assessment of parasite motility was undertaken after 24 hours. Assessing survival only once after a short period of time rendered the assay simpler and shorter. In addition, *T. muris* needs >40 days to develop to the adult stage, a time-frame that is saved while working with L1. Another advantage is that the results of the new assay can be considered more robust given that test worm sample sizes are larger. The assay can be performed using basic laboratory equipment such as a light microscope and an incubator. Compared to the adult motility assay, the total time needed to conduct the assay ought to be as long as half and should not exceed 4 hours spread over 2 consecutive days (for an average of 10 to 15 compounds at a single concentration). Consequently, the total cost for the drug assay can be reduced by approximately 50%. However, a notable drawback of this new method is that it relies on subjective microscopic analysis and includes a relatively time-consuming stimulation step. It is therefore not suitable for high-throughput drug screening.

**Table 1:** **Comparison between the drug sensitivity motility assay for *T. muris *L1 and adult parasites**

	***T. muris *L1 assay**	**Adult *T. muris *assay**
Duration of drug assay^a^	24 hours	72 hours
Number of parasites per well	20 to 200	2 to 4
Dissection of animal host required	No	Yes
Subjective analysis	Yes	Yes
Viability marker alternative	No	Yes^b^
Total operational hours	3.5 hours^c^	6.5 hours^d^
Applicability to high-throughput screening	No	No

^a^: Time between start of the assay and its read-out. ^b^: Resazurin (Alamar Blue^®^). ^c^: Including preparation and reading of the assay. ^d^: Including Infection, keeping and dissection of the animal host, collection of the parasites, preparation and reading of the assay.

Neither resazurin nor the LIVE/DEAD^®^ viability/cytotoxicity kit nor the feeding inhibition assay could provide consistent data about the condition of the larvae and drug effects on *T. muris* L1. Difficulties raised by the presence of the *E. coli* in the culture medium, could be solved to a certain extent by the use of the antibiotic ciprofloxacin (500 μg/mL). However, none of these viability and cytoxicity markers was able to detect a fluorescent signal that would reflect the parasites’ viability and correlate with the findings of the motility assay.

In the resazurin assay, increased concentrations of nitazoxanide led to higher emission values, although not proportionally. This might suggest fluorescent properties of nitazoxanide or DMSO themselves, but this claim cannot be backed by published studies with adult *T. muris*[10]. The feeding inhibition assay, which already failed as a viability marker for *A. ceylanicum* L3 [15], cannot be used as a reliable viability marker for *T. muris* L1 either, since the larvae seem not to ingest significant amounts of fluorescein-albumin. *T. muris* L1 incubated together with ethidium homodimer-1 did not display significant fluorescent emissions. Calcein AM, although significant emission could be measured, did not enable discrimination between viable and drug-treated L1. No correlation was observed between the signal detected and the number of parasites per well. Hence, the Live/Dead^®^ viability/cytotoxicity assay, successfully used in *Leishmania* research [18,19], cannot be applied to *T. muris* L1 viability screening. As documented by Kaneshiro *et al.*[25], the combination of calcein AM and ethidium homodimer-1 shows strongly varying results in yeast and bacteria. Some of the proposed reasons for this are, possible lower esterase activity or the presence of thicker and less permeable outer structures than mammalian cells, for which the assay has been designed.

## Conclusion

In conclusion, we have developed a reliable, cost-effective, time- and labor-saving assay to evaluate drug sensitivity in *T. muris* L1. This assay, based on microscopic assessment of parasite motility, is now well-established at the Swiss TPH and, over the past months, several hundreds of new compounds have been tested against *T. muris* L1. The determination of an appropriate viability marker that would refine this new assay and enable high-throughput drug screening with an objective automated endpoint read-out necessitates further research.

## Competing interests

The authors declare that they have no competing interests.

## Authors’ contributions

JK and LT designed the studies. DW carried out the experiments. DW, JK and LT wrote the manuscript. All authors read and approved the final version of the manuscript.
